# A rare case of advanced testicular seminoma in a 78-year-old man managed successfully with carboplatin based chemotherapy: a case report

**DOI:** 10.1186/1757-1626-1-357

**Published:** 2008-11-28

**Authors:** Nabil Ismaili, Sarah Naciri, Said Afqir, Nawfel Mellas, Imane Bekkouch, Sanaa Elmajjaoui, Ouafae Masbah, Nazih Othmani, Aude Flechon, Jean Pierre Droz, Hassan Errihani

**Affiliations:** 1 Department of Medical Oncology at National Institute of Oncology, Rabat, Morocco; 2 Department of Radiotherapy at National Institute of Oncology, Rabat, Morocco; 3 Department of Urology at Ibnsina Hospital, Rabat, Morocco; 4 Department of Medical Oncology, Centre Léon-Bérard, Lyon, France

## Abstract

**Background:**

Germ cell tumours are uncommon in aged man. We present a rare case of metastatic seminoma of the testis associated with liver and renal insufficiencies in a 78-years-old man managed successfully with carboplatin based chemotherapy.

**Case presentation:**

A 78 years old man admitted with signs and symptoms suggestive of a testicular cancer with alteration of health. Computed tomography of the pelvis and abdomen showed a large retroperitoneal tumour. The diagnosis of seminoma was established from the histological study of the left orchidectomy. At admission, the liver and renal check-up showed liver and renal insufficiencies.

**Conclusion:**

The patient received 4 of carboplatin based chemotherapy with significant improvement in symptoms, and complete radiological response.

## Background

Germ cell tumour is the most common solid tumour in the young adult man but this disease is rare in aged man[1,2]. Pure seminoma is the most common form of testicular germ cell tumour. In this note we present a case of metastatic seminoma of the testis, associated with renal and liver insufficiency's which was managed successfully with carboplatin based chemotherapy.

## Case presentation

**Figure 1:**
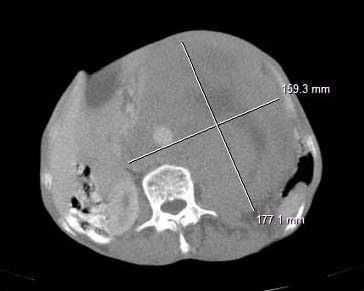
Computed tomography scan of the abdomen showed the retroperitoneal tumour.

A 78 years old man was admitted to our hospital with 4 years history of swollen left testicle. This man suffered from pain in scrotum and abdomen with alteration of health. The performance status at admission was equal to 3 (PS = 3). A physical examination revealed a fixed abdominal mass. The Computed tomography scan of the pelvis, abdomen and lung showed a large retroperitoneal tumour, measuring 18 × 16 cm (Figure 1). The diagnosis of pure seminoma was established from the histological study of the left orchidectomy. The serum markers results showed: AFP (alpha-fetoprotein) = 1,7 ng/ml (normal); β HCG (Human chorionic gonadotropin) = 515 U/L (increased) and LDH (Lactate dehydrogenase) = 3345 U/L (increased). The seminoma was at stage IIC according to the 2002 TNM classification for genitourinary tumours and of good prognosis according to the International Germ Cell Consensus Classification. A complete blood count (red blood cell, white blood cells and platelet) was normal. The estimated creatinine clearance showed renal insufficiency (40 mml/min). The liver check-up showed liver insufficiency with serum transaminase increased up to 2 fold for ALAT and up to 3 fold for ASAT and with GGT (Gamma Glutamyl Transferase) increased up to 2 fold. In addition, the prothrombin rate decreased to 19%. The bilirubinemia was normal. Liver metastasis was the first hypothesis of liver insufficiency considered but the CT scan and ultrasound imaging did not shows liver metastasis. The second hypothesis was the presence of liver infection by B or C virus. This hypothesis was disregarded by the fact that B antibody was positive while HBs antigen was negative. After hydration, corticotherapy and treatment by vitamin K and the increase of PT up to 50% the patient received 2 cycles of chemotherapy with single agent carboplatin AUC 7 with good tolerance of treatment. After the treatment the patient health was drastically improved with normalisation of serum transaminase and prothrombin rate (100%). Then, the patient received 2 other cycles of carboplatin AUC 5 and etoposide (100 mg/m^2 ^at days 1 to 5). After the treatment completed the symptoms disappeared (PS = 0 and the abdomen mass shrunk), the serum markers returned to normal, with complete response of the retroperitoneal tumour in CT scan. The patient is still alive without disease, 5 months after the end of chemotherapy.

## Discussion

Germ cell tumour is the most common solid tumour in the man aged between 15 and 34 years but this disease is uncommon in aged man. Less than 4% of patients with germ cell tumors are aged 65 years or older [1,2]. Pure seminoma is the most common form of testicular germ cell tumour, representing 40–50% of all germ cell tumours. In the elderly patients, 82% of the germ cell tumours were seminomas [3]. The cure rate of germ cell tumour is comprised between 90 to 95%[1]. This success in treatment is the result of multimodality therapy including surgery, radiotherapy and especially cisplatin based chemotherapy [1,4]. Cisplatin is highly effective in combination with other drugs, but universally associated with emesis, neurotoxicity, and renal toxicity. For good-risk patients, etoposide and cisplatin (EP) for four cycles or bleomycin, etoposide, and cisplatin (BEP) for three cycles are the regimens of choice with a durable response rate in excess of 90%[5,6]. Carboplatin, although more myelotoxic does not cause these problems. Initial study suggested a high efficacy for this drug, particularly in seminoma [7,8]. Two series from the Royal Marsden Hospital and Germany evaluated single agent carboplatin in metastatic seminoma. The results from these two studies were remarkably similar, with disease free survival rates of 71% and 77% and survival rates of 91% and 93%, respectively. The medical research council (MRC) randomised 130 patients with metastatic seminoma between carboplatin and cisplatin plus etoposide. Carboplatin was associated with 10% inferior progression-free survival (71 vs 81%) with a non significant survival difference favouring the cisplatin combination (84% vs 89%) [9]. In a multi-institutional study Bajorin et al compared cisplatin and etoposide against carboplatin and etoposide. Twenty-two percent of randomised patients had seminoma. The study showed equivalent response rates and survival but inferior event free survival for carboplatin group [10]. Our elderly patient who developed severe alteration of health, liver and renal insufficiency's, was treated by 2 cycles of carboplatin single agent carboplatin AUC 7 and 2 cycles of carboplatin AUC 5 and etoposide (500 m/m^2^) with significant improvement in symptoms complete response of the retroperitoneal tumour.

## Conclusion

1. The seminoma can arise in the men above the age of 70.

2. Carboplatin in monotherapy or in combination with etoposide can be used to treat elderly patients.

3. Single agent carboplatin AUC 7 can be used to treat advanced seminoma in elderly patients with severe alteration of health, renal and liver insufficiencies.

## Consent

A fully informed written consent was obtained from the patient family for the publication of this case report and accompanying images. A copy of the written consent is available for review by the Editor-in-Chief of this journal.

## Competing interests

The authors declare that they have no competing interests.

## Authors' contributions

All authors have made significant contributions by making diagnosis and intellectual input in the case and writing the manuscript.
